# Simultaneous development of the Pediatric GERD Caregiver Impact Questionnaire (PGCIQ) in American English and American Spanish

**DOI:** 10.1186/1477-7525-3-5

**Published:** 2005-01-14

**Authors:** Jennifer Kim, Dorothy L Keininger, Sara Becker, Joseph A Crawley

**Affiliations:** 1 AstraZeneca LP, Wilmington, DE, USA; 2 Mapi Values, Boston, MA, USA

## Abstract

**Background:**

The objective of this study was to develop simultaneously a new questionnaire, the Pediatric GERD Caregiver Impact Questionnaire (PGCIQ), in American English and American Spanish in order to elucidate the impact of caring for a child with GERD.

**Methods:**

Two focus group discussions were conducted in American English and American Spanish to develop a relevant conceptual model. Focus group participants were the primary caregivers of children with GERD (newborn through 12 years of age). Participant responses were qualitatively analyzed to identify potential differences in caregiver perspectives by the caregiver's language, socio-economic status and demographic profile as well as the child's age and disease severity level. Items in the PGCIQ were generated simultaneously in English and Spanish by reviewing results of qualitative analysis from focus groups in each language. The PGCIQ was finalized in both languages after testing content validity and conducting an in-depth translatability assessment.

**Results:**

Analysis of focus group comments resulted in the development of a first draft questionnaire consisting of 58 items in 10 domains. Content validity testing and an in-depth translatability assessment resulted in wording modification of 37 items, deletion of 14 items and the addition of a domain with five items. Feedback from the content validity testing interviews indicated that the instrument is conceptually relevant in both American English and American Spanish, clear, comprehensive and easy to complete within 10 minutes. The final version of the PGCIQ contains 49 items assessing ten domains. An optional module with nine items is available for investigative research purposes and for use only at baseline.

**Conclusion:**

The PGCIQ was developed using simultaneous item generation, a process that allows for consideration of concept relevance in all stages of development and in all languages being developed. The PGCIQ is the first questionnaire to document the multidimensional impact of caring for an infant or young child with GERD. Linguistic adaptation of the PGCIQ in multiple languages is ongoing. A validation study of the PGCIQ is needed to examine its psychometric properties, further refine the items and develop an appropriate scoring model.

## Background

Gastric reflux of short duration is a normal physiological event for all infants less than six to seven weeks old. When gastric reflux occurs more frequently and past the age of seven weeks, it becomes a clinically significant problem that is diagnosed as pediatric gastroesophageal reflux disease (GERD). Symptoms of pediatric GERD include pain, irritability, frequent spitting-up or vomiting, constant or sudden crying, poor sleep habits and frequent waking. At present, these symptoms represent a clinically significant problem for one of every 500 infants between the ages of six weeks and 18 months [1]. Although these symptoms are not uncommon in childhood, few symptomatic children are treated [2].

The prevalence of symptoms consistent with pediatric GERD varies with age and depends upon the type of symptoms. When referring solely to regurgitation symptoms, over 80% of children experience spontaneous remission by age 18 months [4]. In comparison, findings in the literature indicate that remission of GERD symptoms occurs in 70% of the population at three years of age [8,17]. In fact, research suggests that among children three to nine years of age, only 2.3%, 1.8% and 7.2% will experience symptoms of regurgitation, heartburn and epigastric pain, respectively [18]. Interestingly, despite large variation in prevalence rates by age group, differences in prevalence have not been reported across gender, ethnic groups or socio-economic classes.

Many health care professionals (HCPs) specializing in the treatment of GERD feel that published prevalence rates underestimate the extent of the condition [6,7]. Interviews with HCPs suggest that pediatric GERD is under-treated because many pediatricians are not aware of how to effectively diagnose and treat the condition [8]. Furthermore, many specialists and caregivers believe that GERD is often "missed" by physicians, since it is "normal and common for infants to spit up several times a day" [4]. Failure to properly diagnose, lack of treatment, or sub-optimal treatment of these symptoms can lead to serious complications such as failure to thrive, anemia, esophagitis and respiratory disorders [4].

In addition to having serious consequences for infants and young children, reports from parent advocacy groups suggest that untreated or ineffectively treated pediatric GERD exerts a substantial negative impact on the life of the child's primary caregiver [4]. Caregivers of pediatric GERD patients report sleep loss and psychological and physical strain related to the ineffective or inadequate treatment of pediatric GERD [4]. The burden of care appears to affect every facet of the caregiver's life, including daily activities, social interactions, professional pursuits and family relationships. This burden results in changes in the caregiver's physical and psychological health, quality of life and financial well-being.

The current study had two objectives related to the assessment of the impact of pediatric GERD on the caregiver's daily life. The first objective was to determine if there was an existing instrument suitable for measuring the impact of caring for an infant or child with GERD. If no instrument could be identified, the second objective was to develop an instrument suitable to quantify the impact of pediatric GERD on caregivers, thereby providing a means to improve public awareness of the issue.

### Questionnaire development rationale

A focused literature review was conducted to determine if a caregiver-reported outcome measure that assesses the impact of caring for an infant or child with GERD had been developed. This review included a search of commercial and Mapi Values in-house medical databases of published literature from 1990 to the present in order to identify available instruments and studies relevant to caregiver burden in pediatric GERD. Additionally, the review examined whether generic quality of life measures had been previously applied to the assessment of the impact of caring for a pediatric GERD patient. The literature review uncovered numerous instruments developed for caregivers of adult, elderly or terminally ill patients (i.e., cancer or AIDS patients) [9]. In contrast, few instruments or studies were identified that specifically assess the impact of a child's illness on the primary caregiver. Common approaches to assessing the impact of caring for a chronically ill child included asking caregivers open-ended questions about family strain [10], evaluating the effect of the child's illness on family resources [10] and measuring the impact of the child's illness on the caregiver's well-being and quality of life [11]. No instruments that explore the impact on the caregiver's quality of life of caring for a child with GERD were identified during this review. Nor were any generic measures identified as having been used to quantify the impact of caring for a pediatric GERD patient on the primary caregiver. Furthermore, no single disease-specific instrument was found that assesses the economic, emotional, psychosocial and physical burden experienced by the caregiver of a chronically ill child.

The "Pediatric GERD Caregiver Impact Questionnaire" (PGCIQ) was developed in American English and American Spanish to address the need for an instrument to assess the impact of caring for a child with GERD. This instrument was developed for use in observational studies and multi-national clinical trials to systematically assess and document the physical, psychosocial, psychological and financial impact of caring for pediatric GERD patients. In addition, items in the PGCIQ were specifically developed to capture changes in caregiver burden in response to successful treatment. This instrument will allow documentation of the impact of caring for a child with GERD and provide evidence to increase public and physician awareness of the condition.

The PGCIQ was developed simultaneously in American English and American Spanish to accommodate the rapidly changing population composition of the United States (U.S.). The U.S. Hispanic population grew by 61% from 1970 to 1980 and by another 53% in the following 10 years [12]. Between 1990 and 2000, Hispanics were the fastest growing ethnic group in the country [13]. Because the PGCIQ is intended for use predominantly in the U.S., it was deemed critical that the instrument be sensitive within Spanish-speaking as well as English-speaking populations. Utilizing the simultaneous development approach reduces risk factors that threaten the validity of cross-cultural comparisons in the two language groups.

Although the questionnaire was initially developed in American English and American Spanish, it was designed to be suitable for cross-cultural and linguistic adaptation into multiple languages. Thus, the PGCIQ was developed to provide valuable information in multi-national clinical evaluations regarding the value of different GERD treatments from the caregiver's perspective. Information obtained from the PGCIQ can be used to inform and educate health care providers and payers about the needs of caregivers of pediatric GERD patients.

## Methods

### Simultaneous questionnaire development

The development of the PGCIQ followed the methodology of simultaneous questionnaire development. This process was selected to reduce the risk of systematic measurement error at the item level (i.e., item bias) and ensure that construct bias, which occurs when the construct is measured, was identical across all developed language versions. When combining this methodology with a translatability assessment conducted by linguistic experts, the procedure was intended to yield an "optimal" measure for adaptation of the American English version into different cultures. Additionally, the process aimed to produce a measure that was less susceptible to cultural differences than a questionnaire developed in one language and followed by translation into other languages [14].

### Study participants

Participants were recruited by pediatric gastroenterologists and pediatricians. The primary caregivers included in the discussions were 18 years or older and were caring for children (newborn to 12 years of age) who were either newly diagnosed with GERD or seeking treatment for a new episode of GERD after a period without treatment. Diagnoses of GERD required that children present with common clinical manifestations of GERD. Neonates and young babies were required to have chronic regurgitation, most commonly demonstrated by vomiting. In young children, diagnosis was confirmed by physical discomfort that manifested as prolonged crying, fussing, arching, or refusal of feedings. As well, all children three months or older were required to have symptoms of GERD requiring acid suppressive therapy.

Caregivers were excluded if their child had a history of acute life-threatening events due to manifestations of GERD or a severe unstable illness that could exacerbate caregiver burden. In order to optimize the relevance of the instrument across a wide range of age groups, the study authors attempted to achieve an equal distribution of children within three groups: premature infants to three months, four to 11 months and 12 months to 12 years. Across all three groups, participant eligibility was confirmed by case report forms completed by the treating physician.

### Concept elicitation

Four focus group discussions, two in American English and two in American Spanish, were conducted to elicit issues relevant to caring for a child with GERD. For each language group, discussions were conducted on the East and West Coasts to ensure adequate representation of Mexicans, Puerto Ricans and Cubans, the three largest groups of Spanish-speaking Americans currently residing in the US [15].

Each two-hour focus group was conducted using a structured focus group discussion guide that was developed in American English and linguistically adapted into American Spanish. American English and American Spanish focus groups were facilitated by two female researchers both living in the U.S. The native Spanish speaker was from Ecuador. Focus group moderators received identical, in-depth training from Mapi Values, and all focus groups were videotaped to ensure adherence to the discussion guide. Caregivers were asked to discuss how caring for their child has impacted their lives in the following areas: daily activities, social and family life, emotional and physical functioning and financial well-being. At the start of each focus group discussion, caregivers completed a 48-item GERD Caregiver Informational Survey, developed for this study, which contained questions about the caregiver's and child's demographic, socioeconomic and clinical profile.

**Figure 1:**
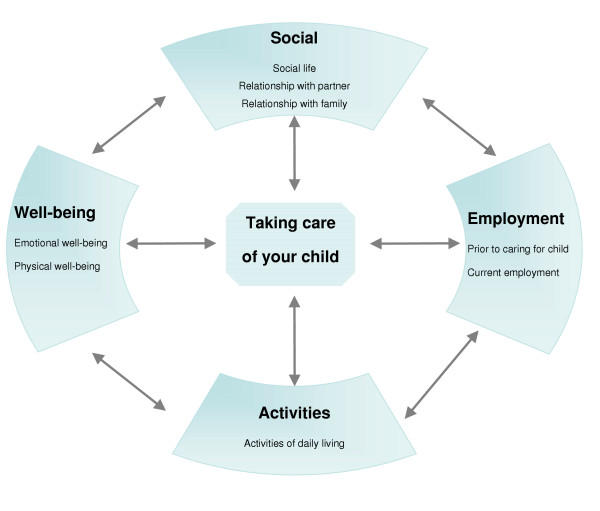
Conceptual Model

Qualitative analysis of the focus group discussions was conducted separately for each language group. This analysis included a comprehensive review of verbatim transcripts by native speaking researchers who also conducted the focus group discussions. Participants' comments were coded to highlight key concepts and psychosocial correlates. Coded comments were subsequently grouped together to elicit the domains and issues important to caregivers. These domains and correlates provided the framework of the conceptual model for questionnaire development (Figure 1).

Following creation of a conceptual model, global concepts within each language group were compared to evaluate similarities and differences in caregiver perspectives. In addition, responses were evaluated to identify potential differences by the child's developmental stage, severity of disease, socio-economic status and demographic profile. Participants' verbatim quotes from each language group were then consolidated within the common domains.

### Item generation

Items were simultaneously generated in American English and American Spanish after consolidating caregivers' verbatim comments. For each item, American English and American Spanish speaking interviewers discussed relevant concepts identified from the focus group discussions and agreed upon conceptually equivalent wording. This process ensured that each item in the questionnaire was pertinent to caregivers in both language groups and formed the basis for Version 1 of the questionnaire.

### Translatability assessment

A panel of linguistic experts conducted a translatability assessment of the PGCIQ to finalize Version 1 of the PGCIQ. The questionnaire was evaluated for its cultural adaptability and translatability in all potential target languages to ensure the cultural equivalence of items. Additionally, this process was used to limit the threat of bias in all current and potential target languages. Items identified as irrelevant for future target languages were considered for deletion or modification in the English and Spanish versions.

### Content validity testing

Content validity interviews were conducted by the native speaking researchers who also conducted the focus group discussions. Researchers interviewed caregivers to assess the ease of comprehension, clarity, cultural equivalence and relevance of the first version of the PGCIQ. Participants were recruited following the same methods and criteria as for the focus group discussions. The interviews aimed to assess the clarity, comprehension and appropriateness of all instructions, questionnaire items and response scales.

Interviews were transcribed verbatim in the participants' native language. Each transcript was comprehensively reviewed, analyzed and coded to highlight caregiver impressions of Version 1 of the PGCIQ. Coded comments were subsequently grouped together to elicit the caregivers' feedback by item. Items were considered for modification if more than one caregiver in either language had difficulties with or suggestions for the item.

The interview results from each language group were analyzed separately and later compared for potential differences. In addition, caregiver responses were evaluated for potential differences by the child's developmental stage, severity of disease, socio-economic status and demographic profile. If an item was identified during content validity testing as potentially problematic, the item was reviewed and simultaneously modified by interviewers in both languages. If an item was problematic in at least one of the languages, the interviewers reviewed the caregivers' verbatim suggestions and attempted to agree upon a conceptually equivalent modification. If no suitable wording could capture the same concept in both languages, the item was deleted from the questionnaire.

The final version of the questionnaire aimed to be relevant to caregivers of pediatric GERD patients, conceptually equivalent in American English and American Spanish, free from bias or jargon and easy to understand, interpret and complete within 10–15 minutes. The final version of the questionnaire was also developed to be appropriate for administration for a fifth grade literacy level, as confirmed by a Flesch-Kincaid Grade level test [17] and linguistic adaptability, as confirmed by a translatability assessment.

## Results

### Focus group discussion participants

#### Caregivers characteristics

Twenty-seven caregivers, 12 American English-speaking and 15 American Spanish-speaking, participated in the focus group discussions. The final distribution of interview participants by geographic region was East Coast English (n = 7), West Coast English (n = 6), East Coast Spanish (n = 5) and West Coast Spanish (n = 9). The average age of focus group participants was 37 years of age with a range of 18 to 64 years of age. The participants were predominantly female (89%), married (81%), living with their spouse and children (89%) and recipients of a high school diploma (81%). The fact that participants were primarily female reflects the composition of the population of primary caregivers seen by the investigators (referring physicians). Moreover, the study investigators attributed the fewer numbers of single and divorced caregivers in the discussion groups to difficulties in taking time off work. Analysis of the caregiver characteristics also revealed differences by language group. Relative to Spanish-speaking participants, a greater proportion of English-speaking respondents were female (100% vs. 80%) and married (100% vs. 67%). Table 1 contains the characteristics of the focus group participants.

**Table 1:** Focus group discussions: caregiver and child characteristics

	**American English n (%)**	**American Spanish n (%)**	**Total n (%)**
**Number of Caregivers**			
by Language	12 (44%)	15 (56%)	27 (100%)
			
**Gender**			
Female	12 (100%)	12 (80%)	24 (89%)
Male	0 (0%)	3 (20%)	3 (11%)
			
**Age**			
Mean	37	37	37
Range	21–58	18–64	18–64
			
**Marital Status**			
Divorced	0 (0%)	1 (7%)	1 (4%)
In long-term relationship	0 (0%)	1 (7%)	1 (4%)
Married	12 (100%)	10 (67%)	22 (81%)
Single	0 (0%)	2 (13%)	2 (7%)
Other	0 (0%)	1 (7%)	1 (4%)
			
**Relationship to Child**			
Aunt or Uncle	0 (0%)	1 (7%)	1 (4%)
Grandparent	1 (8%)	0 (0%)	1 (4%)
Parent	11 (92%)	14 (93%)	25 (93%)
			
**Number of Children**			
by Caregiver Language	12 (44%)	15 (56%)	27 (100%)
			
*Age (years, months)			
Mean	3.6	2.2	2.8
Median	1.6	3.3	2.5
Range	0.2–11.5	0.5–3.6	0.5–11.5
			
**Gender**			
Female	6 (50%)	8 (53%)	14 (52%)
Male	5 (42%)	7 (47%)	12 (44%)
Outlier excluded	1 (8%)	0 (0%)	1 (4%)

*outlier age of 18.2 was excluded in determination of mean, median and range values

#### Children characteristics

The children in this study were primarily female (52%) and their average age was 2.8 years, with a range of six months to eleven years. The average age was determined after excluding an outlier (12 year-olds) from the calculation. In terms of the target age groups, 8% of the sample population was premature up to three month-old infants; 33% were four month to 12 month-old infants; and 55% were 12 month to 12 year-old children. In total, 81% of the children were three years-old or younger, with only five of the children over the age of three. The majority of this sample consisted of children under the age of three years. The age range of this population was consistent with the literature which indicated that the prevalence of GERD symptoms significantly declines with age, with less than 30% of GERD cases persisting past three years of age [17].

According to caregivers' ratings, their children were primarily in very good (26%), good (26%) or fair health (26%). Analysis of additional clinical measures revealed significant differences in the children's health by language group, with Spanish-speakers reporting more hours of fussiness per day, emergency room visits and hospitalizations. Table 1 also contains the characteristics of the children of the focus group participants.

### Concept elicitation

Qualitative analysis of the focus group discussion results revealed nine key concepts relevant to caregivers of pediatric GERD patients: experiences related to GERD diagnosis, taking care of the child, daily activities, emotional well-being, household expenses, physical health, social life, relationships, and employment prior to and after GERD diagnosis. Each key concept is discussed in the following sections.

#### Experiences related to GERD diagnosis

Caregivers recalled feelings of fear, helplessness and guilt associated with the onset of their children's GERD symptoms. Many of these feelings were discussed in the context of negative interactions with HCPs. Caregivers shared stories about their struggle to get doctor appointments or referrals and being told that their children's symptoms were "normal" or that they were "overreacting" or "paranoid." These experiences appeared to be particularly stressful for the Spanish-speaking caregivers. In several cases, Spanish-speaking caregivers stayed with their physicians even though they received inadequate medical attention due to their lack of comfort with the U.S. health system. Thus, their children tended to be diagnosed at an older age than children of English-speakers resulting in elevated feelings of fear, helplessness and guilt. Although caregivers stressed the trauma they experienced in order to obtain their child's diagnosis, this experience did not appear to be change once the child had received an accurate GERD diagnosis. Therefore, this domain will most likely not be affected by treatment and therefore, has been included as optional.

#### Taking care of the child

Caregivers discussed four unique parenting issues related to taking care of a child with GERD: using special feeding techniques, putting the child to bed safely, disciplining the child and finding a reliable childcare provider. First, caregivers of children under four years of age discussed the need to use special feeding techniques. These caregivers commented that they needed to feed their children "small portions," feed them "constantly" and prepare special meals or formulas. Second, caregivers of infants reported adopting unique bedtime routines. These caregivers used equipment such as wedges, car seats, pillows and monitors to put their children to bed safely. Third, caregivers of children over the age of four cited discipline as a challenging issue. Several caregivers recalled emotional tales of disciplining their children for throwing up "on purpose," while others feared that their children would spit up every time they were "yelled at" or "punished." Finally, virtually all of the caregivers cited childcare as a major concern. Most caregivers were unwilling or unable to find childcare providers capable of handling their children's special needs. Even those caregivers who found suitable care providers experienced a sense of distress due to concerns that others would not care for their child adequately. For example, one caregiver stated "No one else is going to give your child the attention [he/she] needs".

#### Impact on daily activities

The caregivers cited three daily activities that were significantly impaired by their children's GERD: mealtimes, housework and bathing. Concerning mealtimes, the caregivers shared stories about spending time "preparing multiple meals" for the family (separate meals for their child), lacking time to "sit down and eat" and having to eat cereal, frozen meals or take-out for dinner multiple evenings in a row. With regard to housework, the caregivers shared anecdotes about having to do laundry "every day," "constantly cleaning the carpet" and putting plastic covers over the furniture to protect it from their children's spit-up or vomit. Finally, the caregivers recalled stories about not having time to bathe, struggling to shower while holding the child and feeling too tired to get dressed. Limitations in these three activities appeared to be most pertinent among caregivers of infants and caregivers of children with more severe GERD symptoms.

#### Impact on emotional well-being

Caring for a child with GERD had a major impact on the caregivers' emotional well-being. The caregivers mentioned a myriad of feelings including fear, worry, grief and depression. The lingering feelings of fear experienced by caregivers most frequently pertained to the possibility that their children might choke and stop breathing. These feelings of fear were reported primarily by caregivers of infants. Feelings of worry were experienced by caregivers of all age groups and predominantly focused on the child's "failure to thrive." These feelings of worry were particularly burdensome for caregivers of children with severe GERD who experienced substantial weight loss and significant developmental delays.

Two specific differences between language groups were found in this domain. English-speaking caregivers mentioned feelings of guilt for "disliking" or "not wanting" their children and feelings of envy upon seeing other "healthy babies." In contrast, Spanish-speakers explained that it was a "given" that caregivers would be close to their children and that they did not experience the feeling of "disliking" their child.

#### Impact on household expenses

Caregivers discussed incurring additional expenses for a variety of products and services related to caring for their children including doctor/hospital bills, special infant formula(s), medicine, laundry, childcare, new or replaced furniture, new or replaced carpets, cleaning supplies and special equipment designed for managing GERD (i.e., wedges, car seats and high chairs). During the focus group discussions, the majority of caregivers spontaneously provided information related to their employment and income level when discussing the financial impact of caring for their children. Where possible, this information was noted and recorded by the moderators for analysis. Additionally, the caregivers were asked information about their insurance coverage and typical expenses in the Caregiver Informational Survey. Analysis of these data indicated that the caregivers' expenses did not differ by their socio-economic status or language group. However, caregivers from lower income households appeared to be more greatly impacted by their additional household expenses and reported greater financial strain.

#### Impact on physical health

The majority of caregivers indicated that their physical health had declined since their children first exhibited symptoms. Common terms used by the caregivers to describe the state of their physical health were "tired," "exhausted," "stressed out" and "distracted." In addition, the caregivers shared numerous stories about carrying their infants all day, sleeping in shifts with their spouses and developing physical ailments such as migraines and skin conditions due to the strain of caring for a child with GERD. These physical health challenges seemed to be particularly burdensome for caregivers of children with more severe cases of GERD.

#### Impact on social life

Caregivers reported reduced social interactions outside of the home and increased social isolation due to their children's GERD. Numerous caregivers commented that they preferred to "stay at home" due to the embarrassment of dealing with their children's constant "vomiting" and "screaming" in public and the emotional strain of seeing healthy babies of the same age as their children. For several caregivers, social isolation was exacerbated by difficulties experienced while talking on the telephone due to their children's "vomiting", "screaming" and need for attention. Issues of social isolation appeared to be most relevant for caregivers of younger infants and caregivers of children with more severe symptoms.

#### Impact on relationships

Caregivers described how caring for their child's GERD affected their relationships with their spouse/partner, relatives, other child(ren) in the family, and their child with GERD. Discussions about the caregiver's relationship with their spouse/partner focused upon the tension experienced between the couple jointly caring for a child with GERD. Tension was often attributed to disagreements over the child's medical care, reduced personal time, reduced desire for physical intimacy and decreased communication. Comments about their relationships with relatives emphasized feelings of frustration with family members who "just don't get it," "complain so much" and "always judge." Remarks about their relationships with other children in the family reflected reduced time to dedicate to the siblings of the GERD patient. Some caregivers confessed that due to the attention required by their child with GERD, they were "less patient," more "demanding" and more "neglecting" of their other children. Finally, stories about the impact on the bond between the child with GERD and the primary caregiver reflected a dual-directional relationship. In some cases, the child's illness seemed to weaken the bond with the primary caregiver, whereas the illness appeared to strengthen the bond in other instances. No major differences were revealed in the impact on these four relationships by language, age, socio-economic or symptom severity group.

#### Impact on employment

The impact of caring for a child with GERD on an individual's employment was primarily dependent on the caregiver's employment situation prior to the child's diagnosis. The caregivers who were previously employed mentioned a number of ways that caring for a child with GERD affected their paid employment including: changes in work schedules, ability to take enough time off to care for their child, reduced productivity and changes in long-term career goals. In order to provide sufficient care for their children, many of the caregivers changed their work schedule by leaving their jobs altogether, switching jobs or cutting back their hours. Moreover, several caregivers took more paid or unpaid vacation time in order to take their children to doctors' appointments, share the burden of care with their spouses or provide more attention to their children. Employed caregivers also described indirect costs in terms of reduced productivity and inability to concentrate at work. Many of those caregivers who were not employed prior to the child's diagnosis reported altering or sacrificing their career plans to ensure they had enough time to devote to the child with GERD. The aforementioned employment issues appeared to be relevant to caregivers across language groups, age groups and symptom severity levels. Overall, however, the employment issues appeared to be especially pertinent for individuals from low-income households, many of whom worked in hourly rather than salaried positions.

### Item generation

Item generation based on nine identified areas and the caregivers' verbatim responses resulted in 10 domains with a total of 58 items that were simultaneously generated in American English and American Spanish. The first draft assessed the following areas: experiences related to diagnosis, caring for the child, daily activities, emotional well-being, physical health, social life, relationships, household expenses and employment prior to onset of GERD and current employment.

The domain assessing the caregivers experiences related to diagnosis was developed for baseline use only. This domain contains six items with "yes" and "no" response options and has a recall period of present time. Issues about employment were divided into two domains pertaining to the time before the child's diagnosis as well as the current time period in order to quantify how the direct and indirect costs associated with caring for a child with GERD change in response to treatment.

With the exception of four domains (experiences related to diagnosis, household expenses, employment prior to onset of GERD and current employment), all questions in the remaining domains have ordinal scales evaluating intensity and frequency. These questions are phrased in the first person, for example: "During the last two weeks, I needed to prepare special meals or formulas for my child." The response format, a 5-point Likert scale was chosen, as the upper limit of an individual's capacity for discriminate judgments has been shown to be near seven (plus or minus two) [18]. Previous studies have suggested that 5-point Likert scales provide an appropriate level for respondents at a fifth grade level of literacy to discriminate without a loss of information. Furthermore, a Likert scale greater than five-points becomes problematic for translation into other languages.

The nine core domains and one optional domain in the first version of the PGCIQ were selected to correspond with the key themes identified from the focus group discussions. To ensure cultural equivalence of items, only those concepts that had been identified in both language groups served as the basis for generating items. Considering that 80% of caregivers had children under the age of three, item generation was predominantly based upon verbatim comments elicited by caregivers of infants and young children.

### Child's age

In the item generation focus group discussions, over 80% of the caregivers had children aged three years or younger. Specific concepts that were noted in the focus group discussions as relevant to caregivers of older children and adolescents, but not to caregivers of infants or toddlers, included discipline issues, concern about the child's emotional well-being and concern about the child's academic development and self-esteem. In contrast, concepts that were relevant to caregivers of infants and toddlers, but not to caregivers of older children or adolescents, included the need to feed the child frequently, feed the child small portions and spend a significant amount of time feeding the child. In general, infants and toddlers also seemed to require more constant one-on-one attention, thereby exerting a greater impact on the caregivers' daily activities, social functioning and family lives.

### Caregiver's gender, education and socio-economic status

The PGCIQ was formulated in caregivers with a range of socio-economic and educational backgrounds. In general, all domains were relevant to caregivers across socio-demographic groups. However, the "employment" and "household expenses" domains appeared more relevant to caregivers of lower economic and educational status due to a higher prevalence of hourly wage earners. Employees working for hourly wages reported greater difficulties getting time off from work to care for their child, less coworker understanding, more financial strain and more pressure to remain in positions they did not enjoy than employees who were salaried. Those in lower income households also struggled more with additional household expenses due to the child's GERD and were more likely to report they could not afford products or services (for example, car seats, wedges, therapy sessions) to improve their children's health.

### Language group

The focus group discussions were conducted in American English and American Spanish. All domains appeared to be relevant to both language groups. Specific issues that were more relevant to the English-speaking groups included experiencing feelings of "envy," feelings of guilt for "disliking the child" and feeling "bonded with the child with GERD." In contrast, employment challenges and issues of tension, reduced intimacy and communication in the partner relationship appeared to be more relevant to the Spanish-speaking groups. Relative to the English-speakers, the Spanish-speakers also voiced the following unique challenges: more financial strain, more pressure to be an "ideal" parent, more difficulty getting attention from English-speaking doctors, less understanding from co-workers and less access to information about GERD.

### Translatability assessment

The results of the in-depth translatability assessment revealed that 16 of 58 items were potentially problematic for future adaptation of the PGCIQ into other languages. These 16 items were reviewed by native speakers. Four of the items were deleted and 12 of the items were modified to improve the cultural adaptability of the PGCIQ. The modified items were flagged for further testing in content validity interviews.

### Content validity interviews

#### Caregiver and child characteristics

Version 1 of PGCIQ was tested in twenty caregivers, 10 English-speaking and 10 Spanish-speaking, who agreed to participate in content validity testing interviews. The final distribution of interview participants by geographic region was East Coast English (n = 4), West Coast English (n = 6), East Coast Spanish (n = 3) and West Coast Spanish (n = 7). The characteristics of the interview population were similar to those of the focus group population. The average age of the participants was 38 years with a range from 24 to 79 years. The majority of the participants were female (70%), married (75%), living with their spouse and children (70%) and recipients of a high school degree (75%). These characteristics resemble those of the focus group population and favor the experiences of married females. Table 2 provides the characteristics of content validity interview participants.

**Table 2:** Content validity interviews: caregiver and child characteristics

	**American English N (%)**	**American Spanish N (%)**	**Total N (%)**
**Number of Caregivers**			
by language	10 (50%)	10 (50%)	20 (100%)
			
**Gender**			
Female	8 (80%)	6 (60%)	14 (70%)
Male	2 (20%)	2 (20%)	4 (20%)
Missing Data	0 (0%)	2 (20%)	2 (10%)
			
**Age**			
Mean	31	47	38
Range	24–37	30–79	24–79
			
**Relationship to Child**			
Grandparent	0 (0%)	3 (30%)	3 (15%)
Parent	10 (100%)	5 (50%)	15 (75%)
Missing Data	0 (0%)	2 (20%)	2 (10%)
			
**Number of Children**			
by Caregiver Language	10 (50%)	10 (50%)	20 (100%)
			
**Child's Age **(years, months)			
Mean	0.7	4.2	2.3
Median	0.5	2.0	0.7
Range	0.4–1.4	0.3–10.5	0.3–10.5
			
**Gender**			
Female	4 (40%)	5 (50%)	9 (45%)
Male	6 (60%)	5 (50%)	11 (40%)
Missing Data	0 (0%)	0 (0%)	0 (0%)

The distribution of children was 45% female and 40% male. An additional 15% of the gender data was missing and is not included in these figures. The average age of the population was 2.3 years, with a range from 0.33 to 10.5 years. As noted, the majority of comments used for item generation of Version 1 pertained to infants and toddlers under the age of three years. Thus, an attempt was made to test the relevance of the PGCIQ in caregivers of older children during the content validity interviews. Despite attempts to recruit caregivers of older children, the final age distribution was skewed towards children 12 months-old and younger. Premature through three month-old infants composed 17%, four month-old to 12 month-old infants composed 56% and 12 month-old through 12 year-old children composed 28% of the final distribution. Similar to the age distribution of the focus group discussions, 85% of the interview participants cared for children through the age of three, with only three of the 20 caregivers having children over three years. Table 2 contains characteristics of the children of the interview participants.

#### Caregiver Impressions

Overall, the respondents had positive impressions of the PGCIQ and described it as "easy to understand" and "easy to answer." The respondents were also satisfied with the recall period, length and the format of the questionnaire. The average time of completion was eight minutes, with a range from five to 15 minutes.

The caregivers suggested a number of revisions to improve clarity. These suggestions, combined with the results of the in-depth translatability assessment, resulted in wording modification of 25 items and deletion of 10 items. The majority of wording modifications were slight changes to enhance the clarity of the items. For example, one item "During the last two weeks, I was limited in preparing meals" was modified to "During the last two weeks, I was limited in preparing meals for my family."

Additionally, several caregivers suggested including a new domain to capture their relationships with family members. In response to this feedback, a new domain, "Your Relationship with Your Family Members," was added. Items in this section used the same wording as the items in the "Your Relationship with Your Partner" section, with the word "partner" replaced by "family member(s)."

#### Final questionnaire

The version of the PGCIQ created after content validity testing contains 49 items assessing 10 core domains: "Taking Care of Your Child," "Your Daily Activities," "Your Emotional Well-Being," "Your Household Expenses," "Your Physical Health," "Your Social Life," "Your Relationship with Your Partner," "Your Relationship with Your Family Members," "Your Employment Prior to Caring for Your Child with GERD," and "Your Current Employment." An additional, optional module with nine items, "Your Experiences Related to Diagnosis," is available for investigative research purposes and for use only at baseline.

## Discussion

The following sections present considerations for the PGCIQ's use and potential limitations of the study. The following topics are addressed: "Your Relationship with Your Family Members"; symptom severity level; child's age and caregiver characteristics.

### Your Relationship with Your Family Members

The new domain, "Your Relationship with Your Family Members," was added following content validity testing interviews based on feedback from participants that additional questions about relationships with family members would provide valuable information. This new domain has not been tested with the wording "family members." As this is a significant change that has not been tested, this domain must be scrutinized during the linguistic validation and psychometric testing process.

### Symptom severity level

The PGCIQ was generated utilizing data from caregivers of children with a range of severity levels. Children with more severe GERD or significant co-morbidities (for example, hiatal hernias or developmental disorders) tended to increase the impact upon the primary caregiver across all nine domains. Conversely, a longer time period since diagnosis tended to reduce the impact on the caregiver. The reduction in impact over time appeared to be the result of two factors. First, many children eventually received successful treatment, which alleviated the GERD symptoms and reduced the strain on the caregiver. Second, the caregivers learned to accept and adapt to their children's condition with time.

### Child's age

Given the age distribution of the study population, the PGCIQ is currently recommended for use in caregivers of pediatric GERD patients, newborn through three years old. Despite attempts to recruit across three age groups, ranging from newborn to 12 years of age, 80% of the focus group participants and 85% of the interview participants cared for a child aged newborn through three years of age. Although these age distributions were consistent with findings in the literature that approximately 70% of GERD cases spontaneously remit after age three, we felt that the majority of data favored infants and toddlers. Further testing of this instrument is therefore recommended in order to consider its use in populations of caregivers of children over the age of three. Researchers interested in extending the PGCIQ to children over the age of three are encouraged to consider the issues relevant to a child's age presented in the results section.

### Caregiver's gender, education and socio-economic status

The focus group discussion and content validity participants were primarily married women. From interviews with physicians, it was found that the majority of caregivers who seek treatment for pediatric GERD patients are women. Even though this instrument has been developed in a primarily female population, the identified caregiver issues appear to span gender roles. The men who participated in the focus group discussions were a small, but vocal minority, who elicited the same core domains as the female participants. Particular items of concern when using the questionnaire in men may be those related to domestic daily activities, given that these items tap more stereotypically female responsibilities. However, if the man is the primary caregiver, then it would stand to reason that he would be responsible for domestic daily activities. Future studies should seek to test the validity of the PGCIQ in larger populations of men and women to ensure that the items are equally relevant across gender roles.

## Conclusions

The PGCIQ has been developed in American English and American Spanish following a rigorous methodology of simultaneous item development for use in observational studies and multi-national clinical trials. The instrument is being linguistically validated from the American English version into several other languages following the appropriate methodology. After linguistic validation and psychometric testing is complete, a theoretical scoring model can be developed, and the PGCIQ may be used in international studies.

Considering the age composition of the focus group and content validity interview population, the PGCIQ is currently recommended for use only in caregivers of pediatric GERD patients, newborn through three years old. Further testing of this instrument is recommended in order to consider its use in populations of caregivers of children over the age of three. Additionally, the study population was predominantly female, married and of moderate to high educational status. However, analysis of the caregivers' verbatim comments suggested that the nine domains were equally relevant to men and women, married and unmarried participants and caregivers of different educational levels. Future tests of the PGCIQ should include large samples of men and women of different marital and educational status to confirm the relevance of these domains.

The PGCIQ is the first questionnaire to document the multi-dimensional impact of caring for an infant or young child with GERD, making it a valuable tool to gather information and quantify the relevant issues and impacts experienced by caregivers of pediatric GERD patients. Information gathered from this tool can be used to inform and educate physicians, managed health care organizations, pharmacists and other health care providers about the needs of caregivers. In addition, items in the PGCIQ were specifically developed to capture changes in caregiver impact in response to successful treatment. Because caregivers mentioned all nine domains as affected by their child's GERD, we conjecture that intervention effects would be associated with changed scores in all nine domains. Thus, the PGCIQ may provide valuable information in multi-national clinical evaluations regarding the value of different GERD treatments from the caregivers' perspective.

This simultaneously generated patient-reported outcome instrument is one of the few examples of simultaneous development identified in the literature. This methodology results in a measure with a reduced risk of factors that might otherwise threaten the successful cross-cultural adaptation of the instrument, making the PGCIQ an appropriate instrument for adaptation into multiple languages.

It is important for the PGCIQ to undergo psychometric testing to develop a final scoring model before the instrument can be used to assess the impact of pediatric GERD on caregivers. Academics and researchers interested in obtaining a copy of the PGCIQ should contact the lead author.

## Authors' contributions

JK Participated in the study design and coordination

DLK participated in the design, coordination and analysis and its interpretation

SB participated in the field work and coordination and analysis and interpretation

JAC conceived of the study and participated in its design
